# An investigation on body weights, blood glucose levels and pituitary-gonadal axis hormones in diabetic and metformin-treated diabetic female rats

**Published:** 2012

**Authors:** Pouya Pournaghi, Rajab-Ali Sadrkhanlou, Shapour Hasanzadeh, Azadeh Foroughi

**Affiliations:** 1 *Department of Basic Sciences, Faculty of Veterinary Medicine, Urmia University, Urmia, Iran; *; 2 *Department of Microbiology, Faculty of Veterinary Medicine, Urmia University, Urmia, Iran.*

**Keywords:** Diabetes, Metformin, Body weight, Hormone, Rat

## Abstract

Diabetes is a metabolic disorder which affects whole body systems including reproductive system. Diabetes is also a contributing factor to infertility. Metformin is one of the most common drugs to control hyperglycemia. In this study, 36 adult Sprague-Dawley female rats (170-210 g) were divided into 3 groups (control, diabetic and diabetic-treated by metformin). In second and third groups, diabetes was induced by streptozotocin injection (45 mg kg^-1^, IP) and the third group was treated by metformin hydrochloride (100 mg kg^-1^ day^-1^, PO) for 8 weeks. Body weights were compared and blood glucose, gonadotropins and sexual hormones were measured. In diabetic group the blood glucose level significantly (*P *< 0.05) increased in comparison with that of control and metformin-treated diabetic rats. The results also revealed that, in the untreated diabetic rats, the mean body weights and pituitary-gonadal axis hormones were significantly (*P *< 0.05) reduced in comparison with the control. Although there were significant (*P *< 0.05) reduction in mean body weights in metformin-treated diabetic rats, reduction in pituitary-gonadal axis hormones was not as sharp as in untreated diabetic rats and only level of progesterone was significantly (*P *< 0.05) reduced in comparison with the control. The results of this investigation revealed that there was a clear relationship between experimental diabetes with body weight and pituitary-gonadal axis hormones, and treatment with metformin relatively restored diabetic complications.

## Introduction

Diabetes mellitus is a serious health problem as based on the World Health Organization (WHO) report, the number of diabetic patients is expected to increase from 171 million in year 2000 to 300 million in year 2025 and 366 million or more by the year 2030.^1^ Diabetes mellitus affects nearly every organ and body system. Diabetes mellitus has adverse effects on sexual function and fertility in women, but the effects on women's sexual health have received limited attention. Between years 1997 to 2002, approximately 1983 articles has been published on diabetes and male sexual dysfunctions, compared to only 13 articles published on diabetes and female sexual dysfunctions. This subject invokes the urgent need for more research in this field.^2^ Some observations suggest an association between the reproductive system, sex hormones, and the development of type I diabetes and related end-organ complications. However, very few studies have actually measured sex hormone levels in patients with type I diabetes, their amounts after treatment with a drug, correlation between development of diabetes with changes in sex hormone levels and its complications.^3^

The methylnitrosourea derivative of 2-deoxy-glucose, streptozotocin (STZ), is a valuable experimental agent used to produce insulin-dependent diabetes in a number of animal species, including rat, which is a good lab model for experimental diabetes. Streptozotocin is a well-known agent for induction of experimental diabetes.^4^

Metformin hydrochloride is a biguanide that is an amino group-rich compound like aminoguanidine used in treatment of diabetes.^5^ Metformin is a rather safe drug and its anti-hyperglycemic property has been generally attributed to combination of a decreased rate of intestinal absorption of carbohydrate, decreased hepatic gluconeogenesis and improvement of peripheral glucose utilization.^6^

The aim of this investigation was to evaluate the effects of experimental diabetes and treatment with metformin on blood glucose levels, pituitary-gonadal axis hormones and body weights. 

## Materials and methods

**Animals. **Adult Sprague-Dawley female rats weighing 170-210 g (10-12 week old) were used in this study. Animals were obtained from Experimental Animal Care Center, Faculty of Veterinary Medicine, Urmia University and were maintained under standard conditions of humidity (45%), temperature (22 ± 2 °C) and light (12 h light/12 h dark), to gain desired body weight. They were fed with a standard rats pellet diet and had free access to water. The animals were randomly divided into 3 groups consisting of 12 rats each; Group C: controls; Group D: STZ-induced diabetic rats; Group M: STZ-induced diabetic rats treated with metformin.

**Drugs and chemicals. **Streptozotocin (Sigma, St. Louis, USA.) was dissolved in freshly prepared sodium citrate buffer 0.1M (pH 4.5) and metformin HCl (Merck Sante S.A.S., Lyon, France) was dissolved in sterile distilled water and administered immediately after preparation.

**Induction of diabetes. **In groups D and M, diabetes was induced by a single intraperitoneal injection of STZ at a dosage of 45 mg kg^-1^ BW in overnight fasting animals. This dosage of STZ has also been employed by earlier researchers to induce diabetes in rats.^7^ Diabetes was confirmed 48 hours after injection of STZ. The blood of fasting animals collected from the tail vein by 24 gauge needles and glucose levels were determined with an automated glucose analyzer device (Glucometer, On Call EZ, SD, USA). Rats with blood glucose levels more than 200 mg dL^-1^ were considered diabetic and included in the study. Control animals were injected with 0.1 M citrate buffer, intraperitoneally. 

**Treatment with metformin. **Oral treatment of metformin HCl (l00 mg kg^-1^, per day by gavage) was started at the 15th day after confirmation of diabetes. The treatment was continued for eight weeks (two four-week periods). The duration of treatment was based on work of others that have used treatment with metformin in four-week period.^8^^-^^10^

**Body weight and blood collection. **At the end of the study (71st day after confirmation of diabetes in groups D and M), the weight of each overnight fasting rat was recorded. Then, they were anesthetized with diethyl ether. For measurement of serum glucose levels, gonadotropins and sexual hormones the blood was taken through cardiac puncture and serum was separated from the blood cells by centrifugation. Then, all serum samples were immediately stored at -20 °C until further analyses. Serum glucose levels were determined by spectrophotometry using glucose oxidase method (Unico 1200, Japan). 

Serum gonadotropins and sexual hormones levels were determined with ELISA using specific diagnostic kits including Follicle Stimulating Hormone (FSH) and Luteinizing Hormone (LH) (DRG Instruments GmbH, Germany); 17-β Estradiol, progesterone, and testosterone (Diaplus Inc., USA). Physiological differences in pituitary gonadal hormones status happens in different stages of sexual cycle. To obtain confidential results from hormonal assays, we divided each group into two sub-groups: in follicular phase and in luteal phase of ovary; according to sex cycle of each rat.^11^

**Statistical analysis. **The data for various parameters (blood glucose, body weight and hormones level) were analyzed using SAS software, version 9.1 (SAS. Institute Inc., Cary, NC, USA). All data were reported as mean ± SEM and to evaluate significant differences, the comparison of means between each two experimental groups was done by Student *t*-test (paired *t*-test). Differences were considered to be statistically significant when *P *< 0.05.

## Results

Symptoms normally associated with the diabetic state such as polyuria, polydipsia and polyphagia were seen in the diabetic rats (in group D more than group M). 

**Fig. 1:**
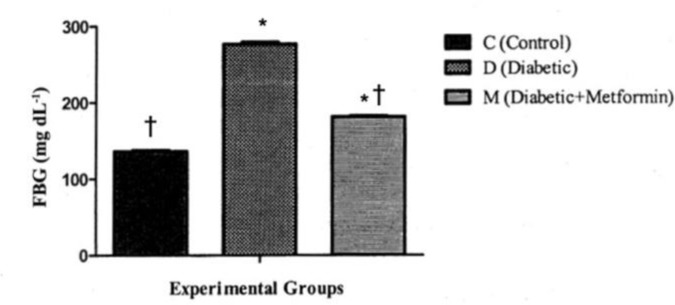
Effect of experimental diabetes and treatment with metformin on FBG. * indicates significant difference at *P *< 0.05 with group C. † indicates significant difference at *P *< 0.05 with group D

**Effects on fasting blood glucose (FBG).** Evaluation of serum glucose revealed that there was significant increase in both D and M groups compared to that of group C (*P *< 0.05). There were also significant differences in levels of the parameters (*P *< 0.05) between groups D and M. The highest and lowest serum glucose levels were recorded in groups D and C, respectively (Fig. 1).

**Fig. 2:**
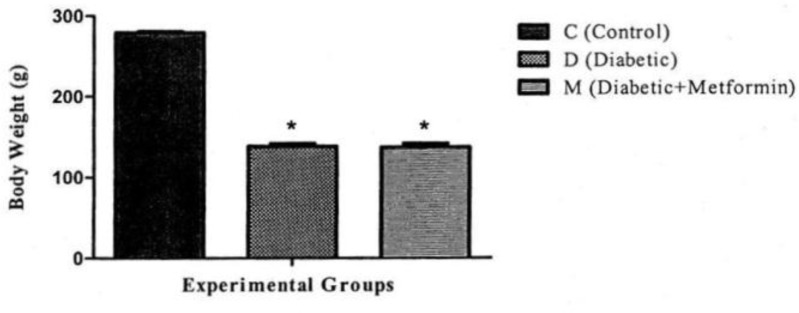
Effect of experimental diabetes and treatment with metformin on body weight. * indicates significant difference at *P *< 0.05 with group C

**Effects on body weight.** There was a significant reduction in mean body weight between group C with D and M groups (*P *< 0.05). However, group M showed a non-significant reduction in mean body weight as compared to group D (Fig. 2).

**Effects on gonadotropins.** Mean ± SEM of FSH concentration showed the significant reduction (*P *< 0.05) in group D compared to group C in both follicular and luteal phases of the ovaries. But FSH concentration in group M displayed no significant difference with groups D and C. Evaluation of LH concentration -in follicular and luteal phases of ovary- showed reduction in both groups D and M compared to group C and a reduction in group D compared to group M. The differences between all paired groups (C&D, C&M and M&D) were significant (*P *< 0.05). These data are displayed on Tables 1 and 2. 

**Effects on sexual hormones.** As shown in Tables 1 and 2, mean ± SEM of 17-β Estradiol concentration showed the significant reduction (*P *< 0.05) in group D compared to group C in both follicular and luteal phases of ovary. But 17-β Estradiol concentration in group M displayed no significant difference with groups D and C. Evaluation of progesterone concentration in follicular and luteal phases of ovary showed significant reductions in both D and M groups compared to group C (*P *< 0.05) and a non-significant reduction in group M in comparison with group D. Total testosterone concentrations in follicular phase of ovary in both groups D and M were significantly higher than those of group C (*P *< 0.05). However, metformin-treated diabetic rats displayed a non-significant increase in total testosterone concentration in comparison with group C in luteal phase of ovary. 

**Table 1.** Effect of experimental diabetes and treatment with metformin on blood hormones level in follicular phase of ovary (Mean ± SEM).

**Parameters**	**Group C** **(n=5)**		**Group D** **(n=5)**	**Group M** **(n=4)**
**FSH (IU L** ^-1^ **)**		0.74 ± 0.03	0.52 ± 0.03*	0.63 ± 0.02
**LH (IU L** ^-1^ **)**		0.29 ± 0.00†	0.18 ± 0.00*	0.22 ± 0.01*†
**17-β Estradiol (pg mL** ^-1^ **)**		5.62 ± 0.24	3.18 ± 0.18*	3.95 ± 0.11
**Progesterone (ng mL** ^-1^ **) **		2.17 ± 0.05	1.42 ± 0.05*	1.37 ± 0.00*
**Testosterone (ng mL** ^-1^ **)**		0.17 ± 0.00	0.25 ± 0.00*	0.23 ± 0.00*

* indicates significant difference at *P* < 0.05 with group C.

† indicates significant difference at *P* < 0.05 with group D.

**Table 2:** Effect of experimental diabetes and treatment with metformin on blood hormones level in luteal phase of ovary (Mean ± SEM).

**Parameters**	**Group C** **(n=7)**	**Group D** **(n=7)**	**Group M** **(n=8)**
**FSH (IU L** ^-1^ **)**	0.51 ± 0.02	0.33 ± 0.02*	0.41 ± 0.01
**LH (IU L** ^-1^ **)**	0.28 ± 0.01†	0.17 ± 0.00*	0.21 ± 0.00*†
**17-β Estradiol (pg mL** ^-1^ **)**	3.01 ± 0.18	1.76 ± 0.08*	2.13 ± 0.05
**Progesterone (ng mL** ^-1^ **) **	4.58 ± 0.12	2.88 ± 0.07*	2.71 ± 0.10*
**Testosterone (ng mL** ^-1^ **)**	0.19 ± 0.00	0.24 ± 0.00*	0.22 ± 0.00

* indicates significant difference at *P* < 0.05 with group C.

† indicates significant difference at *P* < 0.05 with group D.

## Discussion

**Serum glucose.** Increase in serum glucose levels can lead to structural and functional changes in target organs of diabetic patients.^12^ In our study, the blood glucose levels of STZ-induced diabetic rats was significantly higher. These higher blood glucose levels were seen during the whole period of study. The blood glucose levels in untreated diabetics and metformin-treated diabetics were significantly higher than those in normal rats in control group. Reduction in pancreatic β-cell mass is associated with development of diabetes; streptozotocin causes pancreatic β-cell death and thus reduces the population of these cells. Effect of streptozotocin on β-cells leads to development of insufficient production of insulin and consequently, the elevation of blood glucose level occurs.^13^

In our investigation, there were also significant differences between blood glucose levels in untreated diabetics and metformin-treated diabetics. The blood glucose levels in metformin-treated diabetic group were lower than untreated diabetic group (*P *< 0.05). Some of the previous studies have reported that metformin exerts its glucose-lowering (hypoglycemic) effect by suppressing hepatic glucose production.^14^^,^^15^ This drug reduces gluconeogenesis in hepatocytes and amplifies glycogenesis and lipogenesis, reduces intestinal absorption of glucose and increases glucose intake in myocytes and adipocytes.^16^^,^^17^ Metformin can reduce the resistance of target cells to insulin.^18^ A mechanism by which metformin improves the insulin sensitivity in diabetic patients, may be through enhancement in binding of insulin to its receptors in several cell types.^19^ Rossetti *et al.* have reported that metformin treatment of diabetic rats for six weeks leads to a significant improvement in glucose tolerance without any increase in insulin secretion. Our data indicated that metformin HCL treatment reduces the blood glucose levels in diabetic rats that is in agreement with the previous similar studies.^13^^,^^19^^,^^20^ Conversely, the blood glucose lowering effect of metformin is dependent to presence of insulin,^18^ therefore, according to our results it seems that an injection of STZ (45 mg kg^-1^) in rats was not able to degenerate all pancreatic β-cells and so did not lead to a complete lack of β-cell population.

**Body weight.** In our study, the body weight of untreated diabetic group was reduced significantly. This reduction of body weight was also seen in metformin-treated diabetic rats as compared to the normal rats in control groups. This data indicated that treatment of diabetic rats by metformin (100 mg kg^-1^ day^-1^) had no inhibitory effect on body weight reduction in diabetic rats. Our results were in accordance with the results of similar previous studies.^13^^,^^19^^,^^20^ Some studies reported that treatment of diabetic rats by metformin with dose of 25 mg kg^-1^ caused more body weight loss as compared to non-treated diabetic rats.^19^ Previous reports have shown significant weight loss associated with metformin treatment in type II diabetes, compared to sulfonylureas or placebo.^21^^-^^23^ Weight loss during metformin treatment was primarily attributed to loss of adipose tissue and this was explained by effects of different ways of metformin on adipose tissue and muscles.^24^^,^^25^ This reduction of body weight can be due to breakdown of tissue proteins in diabetic rats.^19^^,^^26^ Metformin can reduce the adipose tissue mass in the body.^15^^,^^18^ These actions of metformin can explain the body weight loss in diabetic rats treated with metformin in comparison with untreated diabetic and control rats in present study.

**Hormones level**. In this investigation, the blood 17-β Estradiol levels were significantly decreased in untreated diabetic group and non-significantly in metformin-treated diabetic group in comparison with control group. The differences between untreated diabetics and metformin-treated diabetics were not significant. According to a report, estradiol is associated with diabetes mellitus and altered glucose tolerance.^27^ It has been reported that women with type I diabetes showed reduction^28^ or no change^29^ in circulating estradiol levels compared to non-diabetic women. Experimental models of type I diabetes, namely streptozotocin-induced diabetic rodents, have shown a more consistent hormonal profile characterized by reduced estradiol levels in females.^30^^,^^31^ Findings in experimental models for diabetes are generally in agreement with the data in humans in that Goto-Kakizaki rats exhibit decreased estradiol levels.^32^ According to the present study, metformin treatment of diabetic rats with dosage 100 mg kg^-1^ body weight daily for period of 8 weeks, could not cause a significant increase in blood 17-β Estradiol level. In our study, the total blood testosterone levels in untreated diabetic and metformin-treated diabetic groups significantly increased in comparison with control group, whereas this increase of blood testosterone levels was not significant between these two groups. Androgens are associated with diabetes mellitus and altered glucose tolerance.^27^ Experimental models of type I diabetes, namely streptozotocin-induced diabetic rodents, have shown a more consistent hormonal profile which is characterized by increased testosterone levels in females.^30^^,^^31^ Findings in experimental models are generally in agreement with the data in humans in that female db/db mice exhibit increased testosterone levels.^33^ Endogenous free testosterone is positively correlated with FBG and adiposity.^34^ Our study indicates that treatment of STZ-induced diabetic rats by metformin (100 mg kg^-1^ body weight daily for period of 8 weeks) can decrease the blood testosterone levels more in luteal phase than in the follicular phase of ovary.

The results of this investigation revealed that untreated diabetic group and metformin-treated diabetic group had significantly lower blood progesterone levels in comparison with control group. This indicates that the experimental diabetes cause reduction in progesterone level.^34^ Interestingly the progesterone level in metformin-treated diabetic group was less than untreated diabetic group, whereas LH level in metformin-treated diabetic group is significantly higher than that of untreated diabetic group. Tosca *et al*. reported that metformin-induced stimulation of adenosine 5' monophosphate-activated protein kinase (PRKA) impaired progesterone secretion in rat granulosa cells. According to the present study, metformin treatment of diabetic rats (100 mg kg^-1^ daily for period of 8 weeks) cannot lead to increase in blood progesterone levels; moreover can decrease it. In this study, the assay of blood FSH levels in untreated diabetics showed a significant reduction in levels of this hormone compared to controls. In experimental diabetes reduction in FSH level take place in female rats.^35^ Also, the blood levels of FSH in metformin-treated diabetic rats was lower than that of the control rats. The comparison of blood FSH levels between untreated diabetics and metformin-treated diabetics showed that the metformin with dosage of 100 mg kg^-1^ can improve the blood FSH levels in diabetic rats. According to a report, induction of experimental diabetes in female rats, leads to reduction in LH level.^34^ In our study, the blood LH levels in untreated diabetic and metformin-treated diabetic groups was significantly lower than that of the control group. Whereas, treatment of diabetic rats with metformin caused a significant increase in blood LH levels compared to group D, but did not lead to increase in progesterone level and interestingly that caused decrease in progesterone level. This is in accordance with one previous study that was mentioned above.^35^ Our results about significant decrease in FSH and LH levels in untreated diabetic group compared to control group, are in accordance with the results of similar previous study.^28^

It is concluded that there is a direct relationship between experimental diabetes with blood glucose level, body weight and pituitary-gonadal axis hormones, and treatment with metformin restored diabetic complications. This investigation needs to be completed with other studies in future to find out more about how diabetes affects female infertility, and preventive and or therapeutic measures should be considered using drugs such as metformin in reducing infertility problems following diabetes in females.
